# Percutaneous Coronary Intervention (PCI) Post Out-of-Hospital Cardiac Arrest: A Narrative Review

**DOI:** 10.7759/cureus.71420

**Published:** 2024-10-14

**Authors:** Abdulrahman Kashkosh, Bilaal Yousaf Dar, Sabahat Ahmed

**Affiliations:** 1 Internal Medicine, Calderdale and Huddersfield NHS Foundation Trust, Huddersfield, GBR; 2 Medicine, Faculty of Life Sciences and Medicine, King’s College London, London, GBR; 3 Trauma and Orthopaedics, St George’s University Hospitals NHS Foundation Trust, London, GBR

**Keywords:** cardiovascular disease, coronary artery disease, ecmo, emergency coronary angiography, hemodynamic support, impella device, myocardial infarctions, ohca, out-of-hospital cardiac arrest, primary percutaneous coronary intervention (pci)

## Abstract

Cardiovascular disease is a leading cause of mortality worldwide; therefore, preventing death and improving patient outcomes are a priority. Increasing numbers of patients are surviving hospital admissions after an out-of-hospital cardiac arrest (OHCA). An OHCA has a poor prognosis, and myocardial infarctions (MIs) are the most common cause; hence, the use of emergency coronary angiography and percutaneous coronary intervention (PCI) is an important tool in trying to improve survival. This narrative review explores the role of PCI in OHCA management; understanding angiography findings in OHCA patients offers critical insights into underlying coronary artery disease burden, informing the necessity for PCI. Also, looking at specific subgroups, like females, is essential for equitable intervention access and outcome optimization. Understanding the role of support devices such as Impella and extracorporeal membrane oxygenation (ECMO), which show promise in enhancing outcomes by providing hemodynamic support during PCI and improved overall survival, is linked to better neurological outcomes, highlighting the significance of timely PCI and comprehensive post-PCI care.

## Introduction and background

**Figure 1:**
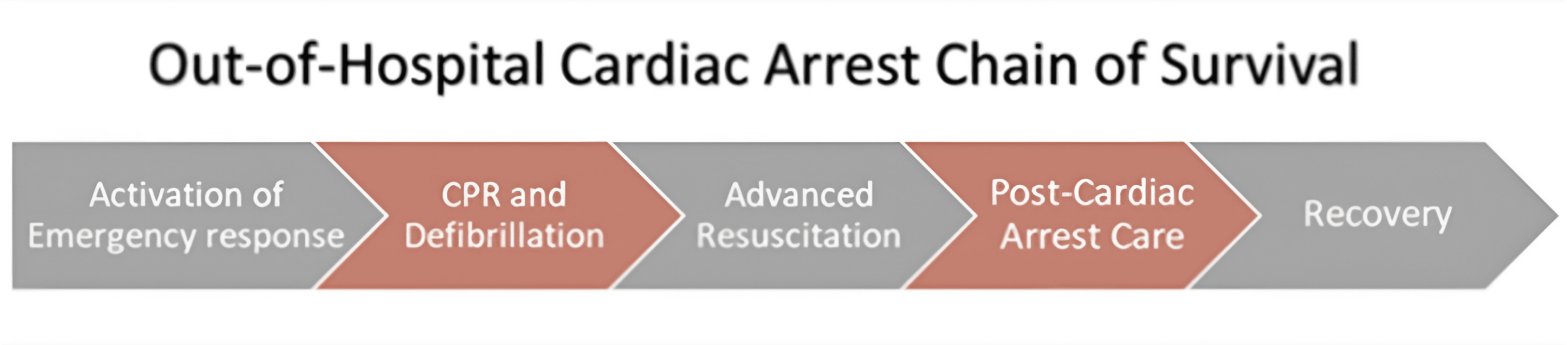
The American Heart Association Chain of Survival This image has been adapted from an article titled *Part 1: Executive Summary: 2020 American Heart Association Guidelines for Cardiopulmonary Resuscitation and Emergency Cardiovascular Care* by Merchant et al. [2]. Image credits: Abdulrahman Kashkosh CPR: cardiopulmonary resuscitation

An increasing number of patients are surviving hospital admissions after out-of-hospital cardiac arrest (OHCA). The increase in bystander cardiopulmonary resuscitation (CPR) awareness and advancements in pre-hospital care contribute to this outcome. The Chain of Survival displayed in Figure 1 highlights the community's importance in the chain's first steps, with patients receiving CPR being increasingly more likely to survive in the past 40 years [1,2].

Yet, for those who survive transportation to the hospital, the outcome remains poor neurologically, with high mortality in hospital and post-discharge. According to the UK National Health Service, an analysis of 30-day survival rates showed it was as low as 9.3% [3]. Out-of-hospital cardiac arrest remains a leading cause of mortality worldwide; thus, preventing death and improving patient outcomes is a priority. Many factors can predict survival and favorable neurological outcomes, ranging from high-quality bystander CPR to post-cardiac arrest care.

## Review

Lack of clarity on angiography and percutaneous coronary intervention (PCI) in OHCA

Out-of-hospital cardiac arrest has a poor prognosis, and myocardial infarctions (MIs) are the most common cause; therefore, the use of emergency coronary angiography and PCI, if indicated, is an important tool in trying to improve survival. Many variables influence outcomes and patient selection for PCI post OHCA. There is a lack of clarity on the benefits and patient selection. Immediate coronary angiography is labor-intensive, costly, and complicated due to limited resources in some centers [4]. There are also risks, such as interrupting the initiation of critical care, leading to poorer patient prognoses, as performing immediate coronary intervention prioritizes coronary circulation over cerebral circulation, delaying the management of possible brain injury. This practice seemingly contradicts the fact that OHCA patients die mainly from neurological complications [5]. Furthermore, numerous risks to patients are added when doing PCI due to the hemodynamic instability in these patients during crucial times. Percutaneous coronary intervention also has intrinsic risks due to exposure to contrast agents, vascular risks, bleeding risks, and stent thrombosis, which are all magnified in these patients [6].

**Figure 2:**
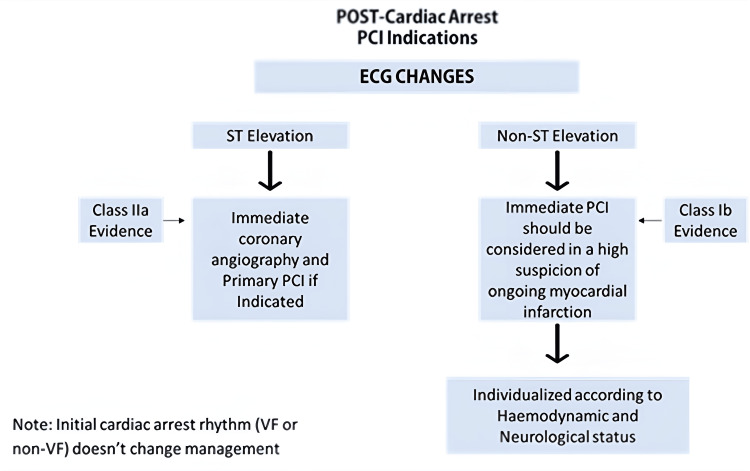
Treatment of PCI post cardiac arrest AHA and ESC guidance summary This image has been adapted from the articles *Early cardiac catheterization is associated with improved survival in comatose survivors of cardiac arrest without STEMI* by Hollenbeck et al. [7] and *Immediate coronary angiography in survivors of out-of-hospital cardiac arrest without obvious extracardiac cause: Who benefits?* by Moutacalli et al [8]. Image credits: Abdulrahman Kashkosh PCI: percutaneous coronary intervention; VF: ventricular fibrillation; AHA: American Heart Association; ESC: European Society of Cardiology

Cardiac arrests are usually spontaneous without any warning and caused by a conduction malfunction, generally leading to an arrhythmia. Most MIs do not result in cardiac arrest, but when a cardiac arrest occurs, a MI is the most common cause. An OHCA can be difficult to gauge as histories, symptoms, and eliciting signs are often complicated by unresponsive patients at admission. An OHCA is usually caused by cardiac causes (although non-cardiac causes can cause it). Other causes, such as arrhythmogenic cardiomyopathies, myocarditis, and genetic disorders, also exist. An electrocardiogram (ECG) is notably the bedside investigation of choice to help determine and screen for the cause of OHCA. Figure 2 summarizes the guidance of the American Heart Association (AHA) and European Society of Cardiology (ESC) on PCI indication post arrest. After OHCA, 40% of patients with ST elevation on ECG were found to have coronary artery occlusion. In non-ST elevation patients, a more varied arrest etiology exists, including non-cardiac causes such as electrolyte disturbances, acute respiratory failure, bleeding, pulmonary embolism, and drug overdose. Nonetheless, even in non-ST elevation, 26.6% of resuscitated patients showed coronary occlusion on immediate angiography [7]. Seventy-five percent of patients referred for immediate coronary angiography post-arrest had significant coronary artery lesions [8].

Pathophysiology of MI leading to a cardiac arrest

Histopathological analysis shows myocardial necrosis is the result of acute MI, typically occurring after 30 minutes of ischemia. Scar tissue forms within the next two months if reperfusion is not established. Acute ischemia causes hypoxia, resulting in intracellular ATP depletion and ADP accumulation. Furthermore, anaerobic respiration products accumulate, leading to intracellular acidosis. This leads to the opening of Na+/H+ channels, followed by Na+/Ca2+ ion exchange channels, resulting in cell swelling and intracellular hypercalcemia. Extracellularly, potassium builds up, resulting in cell membrane depolarization [9]. A reduced gap junction alongside a lower resting transmembrane, followed by intracellular calcium mishandling, causes early and late after-depolarization-induced ventricular ectopics. Ventricular arrhythmias (VA), in particular tachyarrhythmias (ventricular tachycardia (VT) or ventricular fibrillation (VF)), are a leading cause of cardiac arrest in cardiac patients and are common post acute MI [10]. Large MIs increase the risk of more severe myocardial damage and VAs. An OHCA typically occurs late after MI; a previous study found that 6.5 years was the mean interval between MI and OHCA. A left ventricular ejection fraction (LVEF) <30% post MI is also a significant predictor of OHCA [11, 12].

Using tools for clinical assessment and decision-making suitability for PCI

Furthermore, once angiography becomes indicated, deciding which patients have favorable cardiac arrest circumstances is essential to signal suitability for interventions. European and American guidelines suggest favorable cardiac arrest circumstances should signal angiography considerations. Clinical assessment tools for post-cardiac arrest, such as Multivariable Instrument for Neurological Outcome After Cardiac Arrest (MIRACLE_2_), Cardiac Arrest Hospital Prognosis (CAHP), and target temperature management (TTM), are designed to aid the decision-making process; MIRACLE_2_ is particularly promising, as its discrimination performance far outweighs CAHP and performs equal to TTM. Additionally, MIRACLE_2_ is more practical (illustrated in Table 1) than other risk prediction tools. As other prediction tools are complex nomograms, MIRACLE_2_, a quick 10-point score calculation more promising in emergencies. A high MIRACLE_2_ score would indicate a likely poor neurological outcome; hence, cerebral protection becomes a priority, making patients less suitable for the lab. On the other hand, a low score indicates immediate angiography [13-15]. 

**Table 1:** MIRACLE2 score summary The content of this table has been adapted from the articles *A Practical Risk-Score for Early Prediction of Neurological Outcome after Out-of-Hospital Cardiac Arrest− MIRA2CLE2* by Pareek et al. [13] and *The CAHP (Cardiac Arrest Hospital Prognosis) score: a tool for risk stratification after out-of-hospital cardiac arrest* by Maupain et al. [14]. MIRACLE_2_: Multivariable Instrument for Neurological Outcome After Cardiac Arrest

MIRACLE_²_ score	
Variable	Points
Unwitnessed arrest	1
Non-shockable rhythm	1
No pupil reactivity on return of spontaneous circulation (ROSC)	1
Age 60-80 years	1
Age > 80 years	3
Changing rhythm	1
pH < 7.20	1
Any epinephrine dose	2

Early interventions before and during PCI

Undoubtedly, before PCI, many cardiac arrest patients received early interventions. Numerous studies have examined the effect of such early interventions on clinical outcomes. First and foremost, there is a widespread consensus that prompt mechanical CPR significantly improves outcomes in cases of OHCA. Moreover, Anantharaman et al. compared early manual CPR with Lund University Cardiopulmonary Assist System (LUCAS) CPR and found that there was a survival benefit with LUCAS CPR when the device was applied early on-site, demonstrating the benefits of training paramedics on the use of LUCAS to provide a higher quality CPR [16]. Secondly, many cardiac arrest patients receive early intervention with mechanical circulatory support (MCS) before PCI. Basir et al. found that this was associated with improved survival, and centers with protocols highlighting the delivery of early MCS had higher survival [17]. Also, cardiac arrest patients routinely receive mechanical chest compressions (MCC) during PCI and extracorporeal CPR in the cath lab. However, Hardig et al. found that ongoing CPR upon arrival at the cath lab and continued MCC beyond 10-20 minutes in the cath lab were both predictive of poor outcomes [18]. Considering both mechanical circulatory support and early mechanical CPR, Venturini et al. found that the use of MCC during resuscitation of OHCA in the cath lab increased survival and that simultaneous implantation of MCS is safe and practical during MCC-assisted resuscitation in the cath lab [19].

Early angiography

The benefit of early versus late angiography has been a matter of uncertainty. Studies suggest that early angiography (performed within 120 minutes) benefits patients with ST elevation. Zareh et al., in a multicenter study, found that OHCA patients' short-term survival was high with good neurological outcomes when undergoing early angiography and revascularization with TTM [20]. Moreover, Slapnik et al. had similar results in a study looking at the outcome of conscious survivors of OHCA, revealing that conscious survivors of OHCA with ST elevation have excellent survival if they undergo immediate invasive coronary strategy. Since it can be rationalized that conscious survivors of OHCA had no apparent post-resuscitation brain injury, mobilizing them to the cath lab is reasonable. Additionally, the study notes that it is probable that the shorter duration of myocardial ischemia contributed to the positive outcomes [21].

However, for OHCA patients with non-ST elevation, Elfwén et al. showed that early angiography had no advantage compared to late angiography in post-resuscitation myocardial dysfunction parameters [22]. Interestingly, in the same group of patients, it was also found that there was no difference in outcomes whether PCI was performed or not following immediate angiography. This could indicate that patients with non-ST elevation may benefit more from late angiography once they are more hemodynamically stable. In contrast, Kim et al. found that post-resuscitation angiography with and without PCI is associated with better neurological recovery in patients with OHCA, regardless of ECG findings [23]. Additionally, for patients with OHCA due to VF or VT, Vyas et al. looked retrospectively at those successfully resuscitated and concluded that early angiography is associated with higher rates of survival and favorable neurological outcomes [24]. Significantly, a systematic review was carried out by Harhash et al. and found that only a small number of patients with non-ST elevation and a shockable rhythm had angiography. Likewise, data describing the prevalence of coronary artery disease and the outcome of angiography, with or without PCI, in OHCA survivors with non-ST elevation and a shockable rhythm was death [25]. Hanuschak et al. had similar results, concluding that there is a large variability in the use of coronary angiography for OHCA and a lack of uniform practice; future research to determine which patients will benefit most from immediate angiography was recommended [26].

Moreover, Jentzer et al. studied 600 OHCA patients and found that early angiography and PCI are generally associated with improved survival and favorable neurological outcomes. However, after statistical adjustment, early PCI was only associated with a significant benefit. They concluded by advocating immediate angiography in select patients to determine the need for PCI [27]. However, not all medical centers have PCI capabilities, so transporting patients to the nearest PCI-capable center and bypassing the nearest hospital could waste critical time. However, time spent in a non-PCI-capable center before necessitating a transfer could worsen outcomes.

Given that early assessment of occlusion via angiography is associated with better outcomes, it is becoming more accepted that OHCA patients would benefit from direct transfer to centers with PCI capabilities. A retrospective study by Dicker et al. found that 30-day survival was significantly increased in patients transferred directly to a hospital with PCI capability [28]. McKenzie et al. also produced similar results, with patients more likely to survive hospital discharge (adjusted odds ratio 1.97, 95% confidence interval (CI) 1.13-3.43). They also looked at the risk of death up to 12 months afterward and found that indirect transport increased the risk (adjusted hazard ratio 1.36, 95% CI 1.00-1.84) compared to direct transport [29]. Supporting data was also produced by Kragholm et al., who concluded that direct transport to a PCI-capable center is linked with better outcomes regardless of transport time and even when bypassing the nearest hospital [30]. Finally, Tranberg et al. conducted a nationwide follow-up study of over 40,000 OHCA patients in Denmark and concluded that direct admissions to a PCI-capable center were associated with improved survival [31].

Angiography findings in cardiac arrest patients

Several studies have looked into the angiography findings in resuscitated cardiac arrest patients, emphasizing the prevalence of significant coronary artery stenosis and its implications for management and treatment decisions. One notable study by Wester et al. explored the angiography findings in cardiac arrest patients without ST-elevation myocardial infarction. Surprisingly, they discovered that 49.3% of these patients had severe single-vessel disease, indicating the presence of significant coronary artery disease even in the absence of typical STEMI changes [32]. This finding underscores the importance of considering PCI. Further solidifying the role of PCI, Dumas et al. showed that the culprit coronary lesion requiring PCI was found in nearly one-third of OHCA patients without ST-elevation and a two-fold increase in the rate of a favorable outcome [33]. Similarly, a comprehensive systematic review and meta-analysis conducted by Millin et al. revealed that almost one-third of successfully resuscitated cardiac arrest patients without ST elevation exhibited acute lesions on angiography that would benefit from emergent PCI. These findings emphasize the significance of angiography in identifying and managing underlying coronary artery disease [34].

Subgroups 

In addition to analyzing angiography findings among cardiac arrest patients, it is crucial to consider specific subgroups within this OHCA population. One such subgroup is patients with a prior history of cancer. A study by Kang et al. highlighted the disparities in PCI utilization among patients with a previous or current history of cancer, demonstrating a lower probability of receiving potentially beneficial post-resuscitation treatments [35].

Another vital subgroup is female cardiac arrest patients. Jeong et al. found that female patients were significantly less likely to undergo PCI than male patients. Winther-Jensen et al. demonstrated that female cardiac arrest patients were also considerably less likely to undergo angiography, although this was not significantly associated with higher mortality. These gender-based discrepancies highlight the importance of addressing and rectifying any biases or barriers that may exist in the healthcare system. However, it needs to be clarified whether other factors can explain this difference [36, 37].

This disparity in treatment raises essential questions about the influence of various factors, not just previous comorbidities and sex but also race, age, and insurance levels, on the decision-making process. A single-center study by Casey and Mumma found that sex, race, and insurance status were independently associated with post-arrest care interventions, patient outcomes, and treatment [38]. Finally, Aissaoui et al. reported in an extensive French registry of OHCA survivors that early CAG use is associated with a better prognosis. This benefit was persistent up to 75 years, suggesting that age alone should not guide the decision for an early invasive strategy. This study signifies the importance of individualizing care for patients and the need for further research looking at these subgroups to determine if undertreatment is occurring and rectify any biases or barriers in the healthcare system [39].

Support devices

In recent years, support devices have emerged as a potential adjunctive therapy in managing cardiac arrest patients undergoing PCI. These devices aim to provide additional hemodynamic support during the procedure and have shown promising results in improving outcomes.

One such device is Impella, a percutaneous left ventricular assist device (LVAD). A study conducted by Schäfer et al. demonstrated that early utilization of Impella before PCI was associated with improved outcomes. This finding suggests that timely implementation of this support device may improve patient outcomes by enhancing cardiac function and stability during the intervention [40]. Similarly, Loehn et al. and Meraj et al. reported similar positive outcomes associated with Impella use in their study, which looked at MI complicated by cardiogenic shock. Notably, in both studies, patients supported after PCI appear to have poor survival at 30 days. These findings further support the potential benefits of incorporating Impella as part of the treatment strategy for cardiac arrest patients before undergoing PCI [41, 42]. Notwithstanding this, a recent international, multicenter randomized trial, the Danger-Shock trial, found that early use of Impella improved survival in selected STEMI patients without anoxic brain injury. Still, it also led to more non-fatal complications [43].

Another support device that has shown promise in improving outcomes is extracorporeal membrane oxygenation (ECMO). Fu et al. conducted a study highlighting the benefits of ECMO in terms of survival and neurological recovery in cardiac arrest patients. ECMO provides temporary circulatory and respiratory support, allowing for adequate tissue perfusion during the critical period following cardiac arrest [44]. Furthermore, a study by Sugiura et al. explored the weaning process of veno-arterial ECMO (VA ECMO) in post-PCI cardiac arrest patients. They found that the thrombolysis in myocardial infarction flow (TIMI) post-PCI was a predictive factor for successful weaning from VA ECMO support. This finding suggests that monitoring the TIMI flow can guide the decision-making process regarding the duration and discontinuation of VA ECMO support, optimizing patient outcomes [45].

These support devices offer additional hemodynamic support before and during PCI and have demonstrated potential for improving cardiac arrest patients' outcomes. Further research is needed to explore their optimal utilization, long-term effects, and potential combination strategies to enhance patient care and survival rates.

Outcomes

Two important outcomes associated with PCI in cardiac arrest patients are stent thrombosis and neurological outcomes. Understanding these outcomes is crucial for assessing the effectiveness of PCI interventions and optimizing patient care. Stent thrombosis, characterized by the formation of blood clots within coronary stents, is more common in resuscitated cardiac arrest patients compared to other individuals undergoing PCI. This heightened risk emphasizes the need for vigilant monitoring and awareness to reduce this complication, likely improving prognosis [46].

Neurological outcomes are also critical to evaluating the success of PCI in resuscitated OHCS patients. A single-center study has shown that early PCI, dual antiplatelet therapy (DAPT), and better overall survival are associated with improved neurological outcomes [47]. Additionally, Kim et al. retrospectively analyzed around ten thousand OHCA patients and revealed that delayed PCI is associated with worse neurological outcomes. The study concluded that PCI is an advantageous treatment option for all patients with OHCA regardless of established diagnosis [48].

Lastly, Jeong et al. conducted a retrospective analysis on resuscitated OHCA patients with a presumed cardiac cause who underwent successful PCI. They found that patients who underwent delayed PCI for more than 150 minutes after arrest were less likely to achieve neurologically intact survival compared to those who received early intervention. These findings highlight the importance of timely PCI and comprehensive post-PCI care to maximize neurological recovery and minimize long-term deficits [49].

## Conclusions

In conclusion, OHCA typically leads to poor outcomes, with MIs being the most frequent underlying cause. Angiography findings in OHCA patients provide valuable insights into the underlying coronary artery disease burden and help guide the need for early PCI. Understanding these findings and considering specific subgroups, such as females, is crucial for ensuring equitable access to appropriate interventions and optimizing outcomes. Support devices, including Impella and ECMO, have shown promise in improving outcomes by providing hemodynamic support during PCI. Further research is needed to explore their optimal utilization and long-term effects. Stent thrombosis remains a concern in cardiac arrest patients undergoing PCI, emphasizing the need for careful monitoring and appropriate management strategies. Early PCI is associated with improved neurological outcomes, underscoring the importance of timely intervention and comprehensive post-PCI care. Continued research is crucial for guiding decision-making in selecting suitable OHCA-resuscitated patients for early PCI, refining post-PCI treatment strategies, and improving long-term outcomes, given the multiple logistical challenges.
